# IsoMAG—An Automated System for the Immunomagnetic Isolation of Squamous Cell Carcinoma-Derived Circulating Tumor Cells

**DOI:** 10.3390/diagnostics11112040

**Published:** 2021-11-04

**Authors:** Alena Gribko, Janis Stiefel, Lana Liebetanz, Sophie Madeleine Nagel, Julian Künzel, Madita Wandrey, Jan Hagemann, Roland H. Stauber, Christian Freese, Désirée Gül

**Affiliations:** 1 Department of Otorhinolaryngology, University Medical Center Mainz, Langenbeckstr. 1, 55131 Mainz, Germany; algribko@uni-mainz.de (A.G.); sophiemadeleine.nagel@web.de (S.M.N.); wandrey@uni-mainz.de (M.W.); jan.hagemann@unimedizin-mainz.de (J.H.); rstauber@uni-mainz.de (R.H.S.); 2 Fraunhofer Institute for Microengineering and Microsystems IMM, Carl-Zeiss-Str. 18-20, 55129 Mainz, Germany; Janis.Stiefel@imm.fraunhofer.de (J.S.); lana-liebetanz@web.de (L.L.); 3 Department of Otorhinolaryngology, University Hospital Regensburg, Franz-Josef-Strauß-Allee 11, 93053 Regensburg, Germany; julian.kuenzel@ukr.de

**Keywords:** automation, liquid biopsy, circulating tumor cells, head and neck squamous cell carcinoma, immunomagnetic particle-based detection, metastasis

## Abstract

Background: detailed information about circulating tumor cells (CTCs) as an indicator of therapy response and cancer metastasis is crucial not only for basic research but also for diagnostics and therapeutic approaches. Here, we showcase a newly developed IsoMAG IMS system with an optimized protocol for fully automated immunomagnetic enrichment of CTCs, also revealing rare CTC subpopulations. Methods: using different squamous cell carcinoma cell lines, we developed an isolation protocol exploiting highly efficient EpCAM-targeting magnetic beads for automated CTC enrichment by the IsoMAG IMS system. By FACS analysis, we analyzed white blood contamination usually preventing further downstream analysis of enriched cells. Results: 1 µm magnetic beads with tosyl-activated hydrophobic surface properties were found to be optimal for automated CTC enrichment. More than 86.5% and 95% of spiked cancer cells were recovered from both cell culture media or human blood employing our developed protocol. In addition, contamination with white blood cells was minimized to about 1200 cells starting from 7.5 mL blood. Finally, we showed that the system is applicable for HNSCC patient samples and characterized isolated CTCs by immunostaining using a panel of tumor markers. Conclusion: Herein, we demonstrate that the IsoMAG system allows the detection and isolation of CTCs from HNSCC patient blood for disease monitoring in a fully-automated process with a significant leukocyte count reduction. Future developments seek to integrate the IsoMAG IMS system into an automated microfluidic-based isolation workflow to further facilitate single CTC detection also in clinical routine.

## 1. Introduction

Cancer is the second major cause of death in modern society. Besides improved treatment strategies, the development of innovative diagnostics is indispensible for long-term improvement of diagnosis and treatment of cancer patients. The lymph-/hematogenous spread of cancer cells into distant organs and their subsequent growth to overt metastases is the most fatal complication of solid tumors, such as squamous cell carcinoma of the head and neck (HNSCC) [1,2]. Moreover, therapy resistance and associated relapses are common and associated with high patient morbidity [3,4]. The classical view is that metastatic spread is a late process in malignant progression, but today it is accepted that blood circulation and dissemination of primary cancer cells to distant sites is already an early event [5,6].

The fact that these circulating tumor cells (CTCs) are detectable in the peripheral blood of cancer patients months to years after complete removal of the primary tumor in a period called “metastatic cancer dormancy” supports the idea that these cells circulate between metastatic sites [7]. Since their early discovery in the 19th century [8], CTCs have been demonstrated to be clinically recognized and present in the blood circulation of many cancer types including colon [9], lung [10], ovarian [11], breast [12], melanoma [13], prostate [14] and head and neck cancer [15]. Clinical studies present a correlation between the progression of cancer disease and the number of detected CTCs [16,17,18]. A high number of detected CTCs can give information about tumor burden, recurrence and usually represents a poor prognosis. In detail, the determination of CTCs before and after resection also opens up the possibility for monitoring therapeutic response [19] in combination with analyses of circulating tumor DNA (ctDNA) [20]. Hence, the detection and characterization of CTCs as a part of minimal invasive “liquid biopsy” has gained (pre)clinical considerable attention over the last decade [21,22].

However, the capture and detection of CTCs are extremely challenging because of their rarity, the property to move as individual cells or as multi-cellular clumps, and their heterogeneity regarding size and biological and molecular changes during the epithelial-to-mesenchymal transition (EMT) processes. These challenges require the ability to handle a very small number of cells by isolation methods with high efficiency in an acceptable time scale [23]. Various techniques that are based on physical or immunological properties have been developed [24], and different materials with their unique properties according to their shape, size and surface were applied in this field [25,26,27]. Only a few systems are available on the liquid biopsy market but have been shown to isolate CTCs in a proper way. Thereby, these methods often dictate an exact protocol to the user, taking away application flexibility. In contrast to that, researchers aim for process-adaptable platforms as the disciplines of liquid biopsy expand permanently, concerning various sample types such as tissue-derived single cell suspensions or adjustable sample sizes.

All techniques developed for CTC enrichment have their advantages and disadvantages. In contrast to immunomagnetic separation, physical separation methods (e.g., Parsortix^®^ technology, Angle, Surrey, UK) allow label-free isolation but lack cell distinction due to overlapping sizes of CTCs and white blood cells [28]. A microstructure-based enrichment is limited in throughput and sieve-shaped technologies, error-prone to complex liquids such as whole blood, are consequently at risk of clogging in automated lab solutions. Though it has drawbacks in target selectivity, immunomagnetic enrichment is often the method of choice because it is easily automatable and enables a high sample throughput. Since the establishment of the “Food and Drug Administration” (FDA)-approved CellSearch^®^ system, which is held as the gold standard of automated immunomagnetic enrichment and staining platforms, the sensitivity of CTC detection has markedly improved [29,30]. CellSearch^®^ relies on the expression of the epithelial cell adhesion molecule (EpCAM) for the quantification of CTCs in different tumor types [31]. Although this optical detection method have been used in numerous studies, the cost for instruments (220,000 dollars), sample preparations and analysis are still laborious and relatively high (approx. 1000 dollars/run) [32]. Consequently, such assays did not succeed in establishing in clinical routine as a low-cost diagnostic test. Due to the above-mentioned obstacles, fully automated, easy-to-use platforms will demonstrate future solutions to improve cancer diagnostics and therapies as well as basic scientific knowledge of tumor development.

The Fraunhofer CTCelect unit has been developed to meet the need of such a fully automated, easy-to-use platform for CTC isolation out of 7.5 mL whole blood samples. Herein, we characterized the IsoMAG IMS device as the core unit of such a platform applying fully automated immunomagnetic enrichment of rare cells with flexibly selectable antigen targets. In this proof-of-principle study, we established a robust protocol for the automated isolation of an EpCAM- and CSV (cell surface vimentin)-positive subpopulation of cells with a concomitant reduction of background white blood cells as a prerequisite for implementation of the device into the CTCelect unit allowing downstream analyses on a single cell level. Furthermore, we proved feasibility of the method for two squamous cell carcinoma cell lines and HNSCC patient samples. 

## 2. Materials and Methods

### 2.1. Cell Lines

The SCL-1 squamous cell carcinoma line was established by Dr. Petra Boukamp (DKFZ, Heidelberg, DE, Germany) [33] and kept in Gibco™ DMEM (low glucose, pyruvate) medium supplemented with 10% FCS. HNSCCUM-02T squamous cell carcinoma cell line was established by Welkoborsky et al. (UMC Mainz, DE, Germany) [34], and kept in DMEM:F-12 (cc-pro) medium supplemented with 10% FCS and 1% L-glutamine. Cell lines were cultured at subconfluence and incubated at 37 °C in a 5% CO_2_ humidified atmosphere. Other cell lines (A431, HEK293T, MV3, BLM) were received from cell line collections (DSMZ, ATCC) and handled as described before [35,36,37]. 

### 2.2. Blood Samples

All blood samples were obtained from the University Medical Center Mainz, DE. To quantify leukocyte contamination, whole blood bags (500 mL CompoFlex, Fresenius Kabi, Bad Homburg, Germany) from healthy donors were purchased at the local Blood Transfusion Center. Whole blood from HNSCC patients was collected at the Department of Otorhinolaryngology in compliance with ethical guidelines [38].

### 2.3. Immobilization of EpCAM Antibodies on Immunomagnetic Beads

Immunomagnetic beads at different sizes and surface properties (Table 1) were purchased at Thermo Fisher Scientific, Darmstadt, DE and coated with biotinylated monoclonal mouse anti-human EpCAM (CD326) antibody 1B7 (20 µg/mL, eBioscience, GB) or anti-human CSV antibody (clone 84-1, Abnova, Taiwan) for 1 h as described by the manual. Free streptavidin binding sites on anti-EpCAM beads were then saturated with a biotin/PBS solution for 30 min. Anti-EpCAM beads were stored at 4 °C for several weeks.

### 2.4. Manual Cell Isolation by Immunomagnetic Beads

To compare the performance of the IsoMAG IMS unit it was also necessary to establish a standard protocol for manual cell isolation. Cells (SCL-1, HNSCCUM-02T) were released from culture flasks and stained using the CellTrace™ CFSE Cell Proliferation Kit (CFSE) according to the manufacturer’s protocol. A defined number of stained single cells were aspirated with a 10 µL pipette from a 1:100 diluted cell suspension in a petri dish and spiked into 7.5 mL sample volume consisting of cell culture medium, or blood of healthy donors. Next, 100 or 150 µL (depending on medium or blood) of anti-EpCAM Dynabeads MyOne Streptavidin were added (see Table 1) and incubated for 30 min with slow rotation of the tube. Next, the bead-bound cells were magnetically separated for 10 min by using DynaMag-15 (Invitrogen, Thermo Fisher Scientific, Dreieich, Germany) and resuspended in 5 mL washing buffer. Washing steps and magnetic separation were repeated three times using a decreased incubation time of 5 min and resuspension in washing buffer with decreasing volumes (ranging from 5 mL, 4 mL up to 1 mL). 

### 2.5. Automated CTC Enrichment Using the IsoMAG Device

For the establishment of a standard protocol using the IsoMAG IMS unit, antibody-coupled beads were added to a 7.5 mL sample volume consisting of cell culture medium, blood of healthy donors or patient blood samples with spiked EpCAM+ SCL-1 or patient-derived HNSCC cells (HNSCCUM-02T). Before initiating the assay in the software, five tubes containing the input sample and washing buffers were placed in the tube holder carousel and a 10 mL pipet tip was put in the holder of the IsoMAG device as described in Table S1. The sample was incubated for 30 min. After incubation, bead-bound CTCs were magnetically separated alternately with several washing steps in decreasing volumes from 5 mL to 1 mL (see Supplementary Table S1 for detailed protocol).

### 2.6. Characterization of Leukocyte Contamination in IsoMAG Isolates

To quantify analysis-disrupting white blood cell (WBC) contamination in tumor cell isolates, CD45+ cell count after manual and automated IMS was determined by flow cytometry. Therefore, we established a double staining protocol for leukocytes using a CD45-PE antibody targeting the specific surface protein of WBCs (1:50 dilution; Miltenyi Biotec, Bergisch Gladbach, DE, Germany) and nuclear stain RedDot™ (Biotium, Fremont, CA, USA). Wash buffers were set up in the IsoMAG IMS unit and 150 µL EpCAM beads were added to 7.5 mL whole blood before initiating the automated protocol. Manual enrichment was performed in parallel. The final samples were separated in a magnet separator, the supernatant was discarded and the bead cell pellet was stained with the above-mentioned solutions. Dual-positive cells [CD45+/RedDot+] were analyzed by FACS using the BD Accuri C6 flow cytometer. Cell counts after IMS were measured in four samples of different healthy donors and the average WBC contamination with standard deviation (SD) was calculated.

### 2.7. Immunostaining of IsoMAG Isolated CTCs

To assess possible tumor origin of isolated cells, the final 1 mL sample was administered several staining steps. First, isolated cells were stained with fluorescent CD45-FITC antibody (1:1000 dilution) to label leukocytes. Subsequently, the sample was washed three times with PBS in a magnetic separator and transferred to an ibidi 8-well slide (ibidi GmbH, Gräfelfing, Germany) after the last separation step. Cells were centrifuged to the bottom of the slide by a CytoSpin device and fixed in 4% paraformaldehyde for 10 min. After further washing steps, cells were permeabilized (0.1% Triton X in PBS, 5 min) and stained with nuclear dye Hoechst33342 (1:1000 dilution) for 20 min. Following further washing steps, unspecific binding was blocked for 30 min using 0.5% BSA in PBS and staining with fluorescent anti-pan-Cytokeratin-PE antibody (1:200 dilution, overnight) was performed.

### 2.8. Statistical Analysis

Each experiment was repeated at least three times. Data are shown as means with SD and statistical analysis was performed using GraphPad PRISM for Windows (GraphPad Software v9.1.2.226, San Diego, CA, USA, www.graphpad.com, accessed on 13 July 2021). *p*-values were reported as significant when * *p* ≤ 0.05.

## 3. Results

### 3.1. Optimization of Biofunctionalized Beads for Cell Enrichment 

The selection of magnetic beads and immobilized antibodies on the bead surface are decisive factors in terms of cell-specific enrichment. First, EpCAM-biotin antibodies were immobilized on the streptavidin-biofunctionalized beads. To identify a proper CTC model to characterize IsoMAG, manual immunomagnetic enrichment of various epithelial cancer cell lines, such as cutaneous squamous cell carcinoma cells (SCL-1, SCL-2), and A431 was performed (Supplementary Figure S1). We determined the highest recovery rates for EpCAM beads using SCL-1 (85%, *n* = 3) and A431 cells (100 ± 20%, *n* = 3). As controls, we were also able to isolate melanoma cells (MV3, BLM) targeting melanoma-associated chondroitin sulfate proteoglycan (MCSP), but not HEK293T (Supplementary Figure S1B). Being an appropriate squamous epithelial-derived model close to HNSCC, we therefore chose to proceed with the characterization of IsoMAG using the combination of EpCAM beads and SCL-1 cells. For this cell line, high expression of epithelial marker EpCAM is described. EpCAM mRNA expression in SCL-1 cells was confirmed on a single cell level using RT-qPCR (Supplementary Figure S2). 

**Figure 1:**
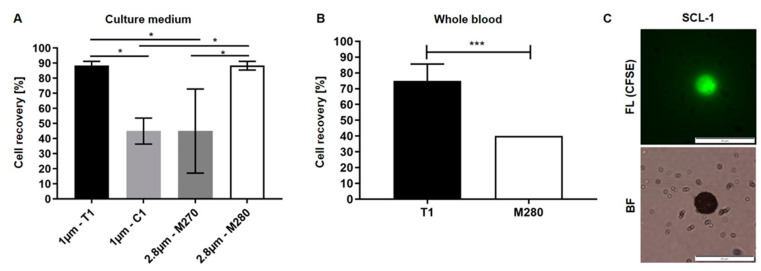
Enrichment of HNSCC cells from cell culture medium (**A**) and whole blood (**B**) differs depending on size and type of functionalized magnetic beads. Values are displayed as means of recovered cells using antibody-coupled magnetic beads with specific sizes and surface modifications. Twenty fluorescently labelled SCL-1 cells were spiked into 7.5 mL cell culture medium (**A**) or 7.5 mL full blood samples (**B**), and manually enriched by using EpCAM antibody coupled magnetic beads. (**A**) Welch’s *t*-test, *n* = 3, *: *p* < 0.05 (one-way ANOVA test with Tukey’s *t*-test); (B) Welch’s *t*-test, *n* = 6 (T1), *n* = 2 (M280), ***: *p* < 0.002; (**C**) recovered, CFSE-stained SCL-1 cells were analyzed by fluorescence microscopy (FL). Corresponding bright-field image (BF) shows beads bound to cell and freely distributed in the medium. Scale bar, 50 µm.

Since there are numerous commercially available beads with different surface modifications and sizes, the next step was to investigate their potential to enrich EpCAM-positive cells spiked into cell culture medium. In this context, four different types of magnetic beads varying in size and surface properties were tested (Table 1). Therefore, 20 fluorescently labeled SCL-1 cells were spiked into 7.5 mL cell culture medium and functionalized magnetic beads were added. After incubation, these cells were manually enriched with a magnetic separator. We obtained the best recovery rates by using 1 µm T1 beads and 2.8 µm M280 beads (Figure 1A). Using these beads, the cell recovery rate was 88.3% (±2.9% SD) whereas the use of 1 µm C1 or 2.8 µm M270 beads resulted in less than 50% cell recovery. 

Subsequently, the performance of T1 and M280 beads was investigated in blood samples of healthy donors. Again, 20 fluorescently labeled SCL-1 cells were spiked into 7.5 mL blood and were manually enriched using the same protocol. As depicted in Figure 1B, the number of recovered cells is significantly higher using T1 beads compared to M280 beads (75 vs. 40%). Microscopic analysis of isolated cells in the counting chamber revealed that cells are densely covered by magnetic beads which are also freely distributed in the enrichment medium (Figure 1C).

**Figure 2:**
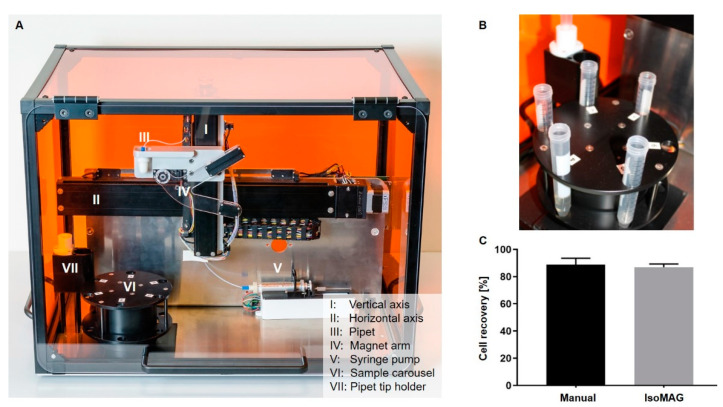
Establishment of automated cell recovery using IsoMAG IMS unit. (**A**) IsoMAG IMS demonstrator with horizontal and vertical axis driving the pipet unit and magnet arm; (**B**) the carousel as tube holder for prestorage of sample and wash buffers; (**C**) 20 fluorescently labelled cells were spiked into 7.5 mL cell culture medium and enriched by using EpCAM antibody coupled T1 (1 µm) magnetic beads. The sample was processed manually or automated using the IsoMAG IMS system. Welch’s *t*-test, *n* = 4, ns: *p* > 0.1234.

Regarding our previous results, T1 beads were chosen for establishment of the automated cell isolation using the IsoMAG IMS demonstrator (Figure 2A). The centerpiece of the benchtop device is a rotatable tube holder carousel with max. 6 reagent slots (Figure 2B). The different positions of the carousel and the 10 mL pipet tip holder to pick up the pipet tip and perform the enrichment are driven by a pipetting robot on a vertical and horizontal axis. The magnet arm is moveable at a swivel joint to capture bead bound cells and free beads in the pipet tip while reducing the sample volume by discarding the supernatant wash buffers. Macrofluidic sample pipetting is directed pneumatically with a syringe pump. The 7.5 mL starting sample is placed into the first position of the carousel. Positions 2–5 are subsequently filled with washing buffers in a decreasing volume and the assay is initiated in the software on an external computer (for detailed protocol see also Supplementary Table S1). 

The results obtained with the IsoMAG IMS unit were directly compared to manually enriched cells with the same batch of magnetic beads. Automated isolation resulted in 86.9% cell recovery while 88.8% of the spiked cells could be detected by manual isolation. This means that in our spiking experiments more than 17 out of 20 cells were recovered from a volume of 7.5 mL by both manual enrichment and the fully automated protocol without significant difference. Statistical analysis of recovery rates showed a slightly lower SD of 2.4% (*n* = 4) after automated isolation compared to manual enrichment (4.8% SD; *n* = 4), indicating a reproducible automation.

### 3.2. Reduction of Blood Cell Contamination

**Figure 3:**
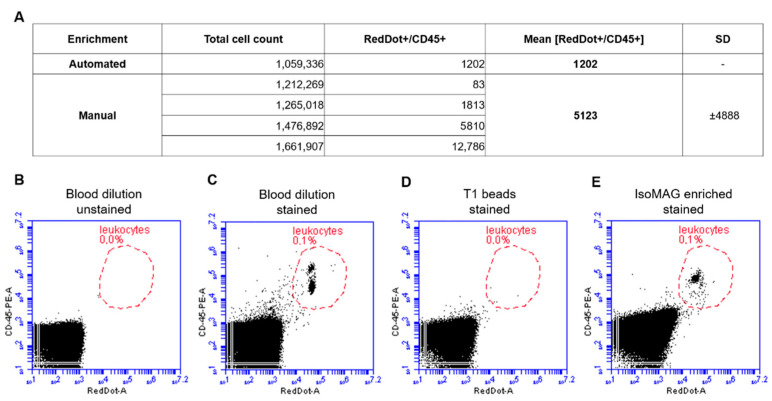
Automated immunomagnetic enrichment procedure exhibits a significantly lower amount of leukocyte contamination compared to manual cell isolation. (**A**) Summary of enrichment processes and quantification of leukocyte contamination. (**B**–**E**) After the automated enrichment process, cells were stained with CD45-PE antibody and nucleic staining reagent RedDot^TM^. Blood dilution (1:1000) shows a dual-positive cell population (**B**,**C**). After the enrichment process, this gating was used to quantify the leukocyte contamination (**E**) while showing no staining of the free beads themselves (**D**).

Besides a robust protocol and an easy-to-handle automated system, it is crucial to reduce the number of “background” WBCs to enable high quality downstream analyses. In preliminary work we already established the basic isolation process, including optimization of necessary washing steps (data not shown). In this study, the automated assay was characterized regarding washing buffers to minimize the unspecific carryover of dual-positive leukocytes [CD45+/RedDot+]. Samples were analyzed by FACS to determine WBC contamination. Gates were set with CD45-PE/RedDot^TM^-stained blood dilution as positive control and stained beads as negative control. The results presented in Figure 3A show that the number of leukocytes after the automated isolation process was about 1200 cells. Comparable results were achieved after manual enrichment (5123 dual-positive cells). However, we observed a broad scattering of WBC counts with a standard deviation of almost 4900 cells in manually enriched samples. Thus, an automated protocol has been developed considering both a high efficiency of cell recovery as well as a low rate of WBC contamination. 

### 3.3. Automated Enrichment of Head and Neck Cancer Cells

We applied the method to a second cancer entity originating from squamous cell tissue with an epithelial, EpCAM-positive phenotype as proof-of-principle. Thus, enrichment experiments were also performed with an epithelial cell line model derived from head and neck squamous cell carcinoma (HNSCCUM-02T). HNSCCUM-02T cells show high EpCAM expression allowing enrichment with our system (Supplementary Figure S3A).

**Figure 4:**
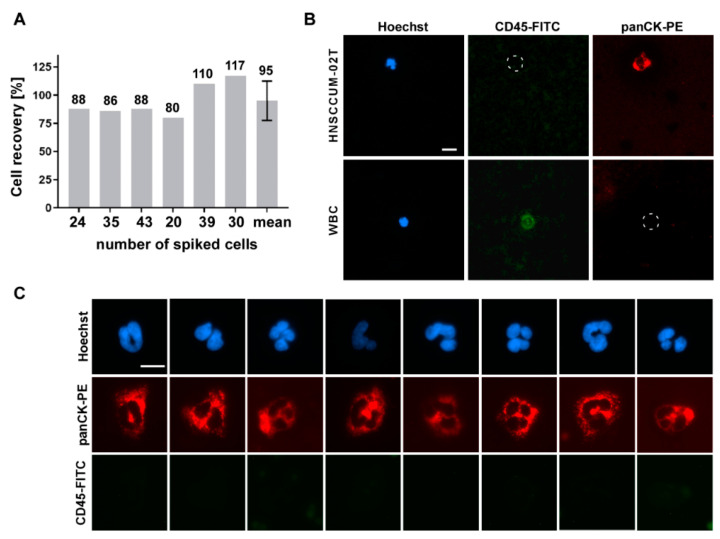
Cell isolation by the IsoMAG System resulted in high recovery rates for head and neck cancer cells. (**A**) Different numbers of HNSCCUM-02T cells were spiked in blood samples and enriched by EpCAM-coupled T1-beads using the IsoMAG unit. (**B**) For cell counting, recovered cells were stained with Hoechst dye, cytokeratin (panCK-PE) and CD45-FITC antibodies and quantified by fluorescence microscopy. [Hoechst+/CD45−/panCK+) cells were classified as HNSCCUM-02T cells, [Hoechst+/CD45+/panCK−] cells as leukocytes (WBC). Scale bar, 10 µm. (**C**) Examples of cells enriched by CSV-coupled T1-beads. Cells were stained as described in (**B**). Scale bar, 10 µm.

**Figure 5:**
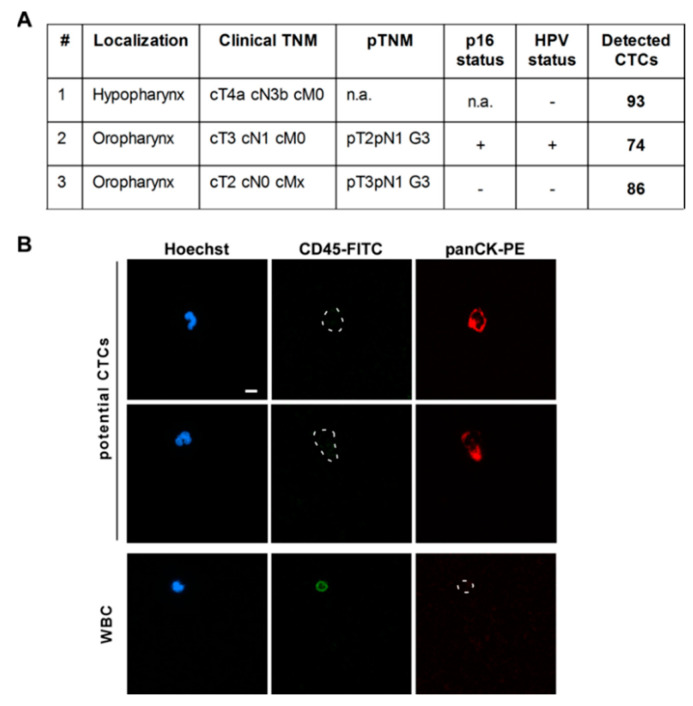
Potential circulating tumor cells (CTCs) could be enriched from head and neck cancer patient blood. (**A**) Clinical parameters and numbers of detected CTCs for three patients suffering from hypo- or oropharyngeal cancers including lymph node metastases. (**B**) 7.5 mL of patient blood was mixed with EpCAM-coupled T1 beads and placed into the IsoMAG unit for automatic enrichment of CTCs. For cell counting, enriched cells were stained with Hoechst dye, cytokeratin (panCK-PE), and CD45-FITC antibodies and quantified by fluorescence microscopy. [Hoechst+/CD45−/panCK+] cells were classified as potential CTCs, [Hoechst+/CD45+/panCK−] cells as leukocytes (WBC). Scale bar, 10 µm.

Automated enrichment (spike-in) experiments with HNSCCUM-02T cells were conducted as described before and demonstrated applicability of the IsoMAG IMS unit using blood samples, both from healthy donors and patient blood (Figure 4 and Figure 5). In total, 95% (±15) of spiked cells were recovered (Figure 4A), quantified by fluorescence microscopy after intracellular staining of epithelial tumor marker pan-cytokeratin (pan-CK) [39] and CD45 (Figure 4B). Isolated tumor cells were identified by expression of cytokeratin [Hoechst+/CD45−/panCK+] and could be differentiated from CD45-positive leukocytes [Hoechst+/CD45+/panCK−] appearing at low frequency after enrichment.

As dynamic EMT and MET processes seem to modulate primary tumors, metastases and CTCs disease- and patient-dependently, detection systems allowing the use of variable CTC/cancer markers are highly desirable. To underline the advantage and full flexibility of our device to also isolate cells by using variable cancer markers, we here targeted cell-surface vimentin (CSV) as an additional proof-of-concept example. As shown in Figure 4C, we performed spike-in experiments using engineered CSV-coupled immunomagnetic beads to isolate HNSCCUM-02T cancer cells from human blood. Expression of CSV was verified by Western blot analysis and immunofluorescent staining (Supplementary Figure S3C,D). Importantly, automated isolation of spiked HNSCCUM-02T cells using CSV-coupled beads also resulted in high recovery rates of ±95% (Figure 4C).

### 3.4. CTC Screening Using Blood of HNSCC Patients

Finally, three HNSCC patients were screened for CTCs applying our established protocol for the IsoMAG IMS system. The results shown in Figure 5 impressively demonstrate the clinical applicability of the system. All cells positive for cytokeratin but negative for CD45 expression [Hoechst+/CD45−/panCK+] were counted and classified as potential CTCs (Figure 5B). Here, we were able to isolate 74 to 93 potential CTCs with an epithelial-like character from patients suffering from different head–neck tumor subtypes (hypo- and oropharynx cancers with lymph node metastasis, see Figure 5A). We also captured preliminary data on isolation of CTCs out of patient blood using a combination of EpCAM and CSV beads (Supplementary Figure S4). However, these results obtained from a small and heterogeneous group of patients are preliminary and have to be confirmed by larger studies and further analysis methods, e.g., sequencing.

## 4. Discussion

Personalized cancer therapy will benefit from analysis of single solid and circulating tumor cells in the future. In order to develop a fully automated system for the enrichment and isolation of single tumor cells, the reduction in sample volume plays a pivotal role. Thus, excellent performance is required for the enrichment step depending on the developed system but also on the developed protocol. 

In the present study, we successfully established a robust protocol for the automated isolation of CTCs by the IsoMAG IMS device, a core unit of the CTCelect platform. As a first step, we developed a suitable CTC model system to establish the protocol for squamous cell carcinoma. Although we also achieved high recovery rates for the epithelial A431 cells, we decided to use SCL-1 and HNSCCUM-02T cell lines as squamous epithelial-derived models close to HNSCC. Of note, we are aware of variable EpCAM expression in “primary CTCs” versus established cancer cell lines. However, for the development and comparison of CTC selection procedures and devices, CTC models with defined EpCAM levels are mandatory. Using this model, we demonstrated improved performance of 1 µm tosyl-activated, hydrophobic magnetic beads (T1) which thus were chosen for our final protocol. In addition to their advantageous characteristics for automated applications, such as a low sedimentation rate and faster reaction kinetics compared to M-280/M-270 beads, our results are in line with other studies showing improved capture efficiency and specificity of 1 µm T1 beads for magnetic cell detection [40,41]. 

Downstream analyses of isolated cells by fluorescence microscopy not only confirmed expression of relevant cancer markers, but also revealed tight binding of magnetic beads to the cell surface (see also Figure 1C). Regarding the size of the used T1 beads (1 µm) and an average diameter of CTCs between 10–12 µm [28], the surface of a cancer cell could theoretically be covered with 300–450 magnetic beads suggesting severe consequences for cell integrity and viability. However, we observed that isolated CTCs are not completely covered with beads, and preliminary studies revealed that re-attachment and cultivation of isolated cells is not excluded, albeit very challenging (see Supplementary Figure S5). Here, additional washing and bead detachment steps, as well as careful adjustments of cell cultivation, such as the use of preconditioned medium and collagen-coated slides, are necessary to receive viable and proliferating cells. Due to the highly versatile construction and design of the device, it is possible to implement such additional purification steps to the automated IsoMAG protocol. Moreover, the biocompatibility of micro/nanocarriers may be affected by biomolecule corona formation [26,42,43]. It is accepted that when micro/nanocarriers enter physiological environments, proteins and other biomolecules bind to their surfaces, leading to the rapid formation of a biomolecule corona [44]. The corona may be critically co-defining the biological, medical, biotechnological and pathophysiological identity of micro/nanocarriers, although the mechanistic details have not been resolved in detail [45,46]. 

As an additional relevant factor, reduction of background white blood cells is mandatory for the establishment of a reliable protocol allowing downstream single-cell analysis. In summary, our study shows that using the optimized protocol for IsoMAG tumor cells from patient samples can be enriched and detected in a semi-automated process combined with a reduction of white blood cells (WBC) starting from a large volume (7.5 mL). The broad scattering of WBC counts we observed could depend on a naturally different blood cell count between healthy individuals. It is conceivable that the WBC count could be a hindrance to nucleic acid-based downstream applications at this point due to enforced background noise. A purity of at least 50% is recommended for proper genomic analysis, whereas in our small patient screening, a vast percentage of total output cells was WBCs. On an average, we were able to isolate 84 potential CTCs from three patient samples, while EpCAM-based enrichment entailed a bycatch of ~1200 WBCs. Nevertheless, the relatively low number of 10^3^ WBCs (compared to 10^7^ WBCs/mL in healthy adults) enabled immunofluorescent assessment of the patient isolates and marker-based distinction between potential CTCs and WBCs to deliver a ready-to-use assay in an otorhinolaryngology lab environment. In comparison, a similar WBC bycatch of over 800 cells/sample was described for CellSearch^®^ [47], whereas size-based Parsortix^®^ delivers purity grades ranging from 29–97% depending on the study [48,49].

To address downstream analysis, subsequent microfluidic cell sorting of the IsoMAG isolates, manual cell picking or aspiration technologies such as the ALS CellCelector™ are appropriate tools to improve signal-to-noise ratio for transcriptomic analysis. We already started implementing IsoMAG in an in-house developed microfluidic single-cell-sorting workflow resulting in a purity of ≥75% (Supplementary Figure S6). By aspirating only dispensed droplets that contain fluorescent cells for RNA isolation, we obtained promising PCR results detecting even small numbers of EpCAM+ tumor cells with low WBC background signal. These findings underline that (immunomagnetic) pre-enrichment of the CTCs by IsoMAG is an indispensable prerequisite for microfluidic cell sorting and downstream CTC analysis. 

Interestingly, during protocol establishment we observed cell recovery rates of >100%. This was likely due to false positive panCK or false negative CD45 staining which has been optimized during establishment. However, our automated protocol combines both a high efficiency of cell recovery as well as a low rate of WBC contamination.

Furthermore, in the presented study we could also show that the established method is adaptable to the detection of EpCAM-, as well as CSV-positive subpopulation of CTCs. Typically, immunomagnetic cell isolation devices use epithelial markers, mostly EpCAM, for CTC detection neglecting the fact that cancer cells undergo morphological changes during epithelial-to-mesenchymal transition (EMT) and the reverse process (MET). Importantly EMT/MET which takes place during entry and transport in the blood stream is accompanied by up- and downregulation of surface markers used for CTC detection, such as EpCAM, N-/E-Cadherine, and cell-surface vimentin [50]. Thus, in contrast to other commercial systems we are able to capture cells exhibiting not only epithelial, but also mesenchymal and/or an intermediate phenotype. Preliminary data combining beads targeting EpCAM as wells as CSV for automated cell enrichment were also promising but have to be confirmed in larger studies to assess the added value of using multiple markers for HNSCC. 

In our small patient screening study, we were able to isolate 74 to 93 potential CTCs with the optimized protocol. Previous studies applying different methods for CTCs detection in HNSCC revealed a broad range of enumerated cells. Whereas Grisanti et al. detected 0–2 CTCs in 16–40% of the patients using the CellSearch^®^ platform [51], manual enumeration of immunofluorescent stained cells resulted in higher CTC numbers (0–37 CTCs/1000 PBMC) [18]. Despite the broad ranges of detected CTCs, the obtained results of our IsoMAG IMS unit are in the order of previous studies combined with low numbers of leukocyte contamination. We could also observe a slight tendency for an increasing CTC number in T4 staged tumors compared to T1–3 as described previously [16,18]. Of course, these results have to be interpreted with caution because the small number of patients does not allow reliable assessment. This observation and the fact that a high mortality rate of HNSCC patients correlates to late diagnosis show the necessity of an early and reliable detection method of CTCs. Semi-automated detection of CTCs such as IsoMAG has the potential to be related as a standardized part of liquid biopsy with the advantages of real-time personalized analysis in combination with non-invasiveness and individual prognostic therapy [15].

Taken together, there are several findings of this study underlining the advantages of the IsoMAG device compared to other immunomagnetic isolation methods. Whereas CellSearch^®^ is marketed relying only on epithelial targeting of preserved cells in the CELLTRACKS^®^ AUTOPREP^®^ System and requires centrifugation [52], the IsoMAG protocol allows highly flexible targeting of viable CTCs without blood pre-processing. Implementation into the CTCelect workflow in future will allow downstream single-cell analysis and cell cultivation. The general major advantage of automated cell isolation in contrast to manual enrichment consists of standardizing protocols and preventing human errors while reducing costs and hands-on time. Commercial magnetic cell separation devices, i.e., an autoMACS^®^ Separator (Miltenyi Biotec, Bergisch Gladbach, Germany) were strongly improved over the last few years but were mostly marketed to isolate abundant cell populations, rather than rare cells such as CTCs. With regard to the long-term goal of a low-cost diagnostic test, IsoMAG was characterized with in-house functionalized EpCAM beads minimizing material expenses to 35 EUR per 7.5 mL blood compared to 102 EUR for Miltenyi StraightFrom^®^ Whole Blood CD326 MicroBeads. Conclusively, the overall aim of our study is the implementation of IsoMAG IMS unit as a core unit of the CTCelect for the fully automated isolation of single CTCs without sample preparation. Due to urgently needed low cost minimally invasive diagnostics methods in the clinical routine, allowing the sensitive and reliable detection of tumor components in patients’ blood, the establishment of our IsoMAG IMS unit of CTCelect also represents a technology for single cell isolation and comprehensive downstream “omics”-based analyses. Such approaches will deepen our understanding of CTC pathobiology, a prerequisite for improved treatments and management of cancer patients in the future.

## 5. Conclusions

Here, the automated IsoMAG IMS unit was thoroughly characterized as a reliable, economic, and straightforward technology for automated and reproducible CTC detection and enrichment. In addition, the IsoMAG IMS system was shown to allow the use of variable cancer/CTC markers, such as cell surface vimentin. Its flexibility can be tailored to the user’s specific needs for various malignancies and/or cell types. Future developments aim to combine immunomagnetic separation with microfluidic devices to further improve the power of automated immunomagnetic cell isolation devices for research and the clinics.

## Figures and Tables

**Table 1:** Overview of tested Dynabeads.

Beads	Size	Surface Functionalization
Dynabeads MyOne Streptavidin T1	1 µm	Tosyl-activated, hydrophobic
Dynabeads MyOne Streptavidin C1	1 µm	Carboxylic acid, hydrophilic
Dynabeads M-270 Streptavidin	2.8 µm	Carboxylic acid, hydrophilic
Dynabeads M-280 Streptavidin	2.8 µm	Tosyl-activated, hydrophobic

## Data Availability

The data presented in this study are available on request from the corresponding authors.

## Supplementary Materials

The following are available online at https://www.mdpi.com/article/10.3390/diagnostics11112040/s1: Figure S1: Recovery rates of different cancer cell lines; Figure S2: Gel electrophoresis of EpCAM RT-qPCR products from SCL-1 single cell RNA; Figure S3: Expression of EpCAM protein (A and B) and CSV (C and D) in HNSCCUM-02T; Figure S4: Potential CTCs could be enriched from whole blood of a HNSCC patient using EpCAM- and CSV beads; Figure S5: Brightfield microscopic images of HNSCCUM-02T cells cultivated after immunomagnetic isolation; Figure S6: Establishment of CTCelect unit improves purity of isolated tumor cells from whole blood; Table S1: Detailed protocol of IsoMAG isolation procedure.

## References

[1] Kunzel J., Gribko A., Lu Q., Stauber R.H., Wunsch D. (2019). Nanomedical detection and downstream analysis of circulating tumor cells in head and neck patients. Biol. Chem..

[2] Gül D., Habtemichael N., Dietrich D., Dietrich J., Gößwein D., Khamis A., Deuss E., Künzel J., Schneider G., Strieth S. (2021). Identification of cytokeratin24 as a tumor suppressor for the management of head and neck cancer. Biol. Chem..

[3] Siemer S., Fauth T., Scholz P., Al-Zamel Y., Khamis A., Gül D., Freudelsperger L., Wollenberg B., Becker S., Stauber R.H. (2021). Profiling Cisplatin Resistance in Head and Neck Cancer: A Critical Role of the VRAC Ion Channel for Chemoresistance. Cancers.

[4] Beltz A., Gosswein D., Zimmer S., Limburg I., Wunsch D., Gribko A., Deichelbohrer M., Hagemann J., Stauber R.H., Kunzel J. (2019). Staging of oropharyngeal squamous cell carcinoma of the head and neck: Prognostic features and power of the 8th edition of the UICC staging manual. Eur. J. Surg. Oncol..

[5] Kiweler N., Wunsch D., Wirth M., Mahendrarajah N., Schneider G., Stauber R.H., Brenner W., Butter F., Kramer O.H. (2020). Histone deacetylase inhibitors dysregulate DNA repair proteins and antagonize metastasis-associated processes. J. Cancer Res. Clin. Oncol..

[6] Hu Y., Yu X., Xu G., Liu S. (2017). Metastasis: An early event in cancer progression. J. Cancer Res. Clin. Oncol..

[7] Park S.-Y., Nam J.-S. (2020). The force awakens: Metastatic dormant cancer cells. Exp. Mol. Med..

[8] Ashworth T.R. (1869). A case of cancer in which cells similar to those in the tumours were seen in the blood after death. Australas. Med. J..

[9] Murray N.P., Albarran V., Perez G., Villalon R., Ruiz A. (2015). Secondary Circulating Tumor Cells (CTCs) but not Primary CTCs are Associated with the Clinico-Pathological Parameters in Chilean Patients With Colo-Rectal Cancer. Asian Pac. J. Cancer Prev..

[10] Wendel M., Bazhenova L., Boshuizen R., Kolatkar A., Honnatti M., Cho E.H., Marrinucci D., Sandhu A., Perricone A., Thistlethwaite P. (2012). Fluid biopsy for circulating tumor cell identification in patients with early-and late-stage non-small cell lung cancer: A glimpse into lung cancer biology. Phys. Biol..

[11] Engel H., Kleespies C., Friedrich J., Breidenbach M., Kallenborn A., Schondorf T., Kolhagen H., Mallmann P. (1999). Detection of circulating tumour cells in patients with breast or ovarian cancer by molecular cytogenetics. Br. J. Cancer.

[12] Jaeger B.A., Jueckstock J., Andergassen U., Salmen J., Schochter F., Fink V., Alunni-Fabbroni M., Rezai M., Beck T., Beckmann M.W. (2014). Evaluation of two different analytical methods for circulating tumor cell detection in peripheral blood of patients with primary breast cancer. Biomed. Res. Int..

[13] Mocellin S., Hoon D., Ambrosi A., Nitti D., Rossi C.R. (2006). The prognostic value of circulating tumor cells in patients with melanoma: A systematic review and meta-analysis. Clin. Cancer Res..

[14] de Bono J.S., Scher H.I., Montgomery R.B., Parker C., Miller M.C., Tissing H., Doyle G.V., Terstappen L.W., Pienta K.J., Raghavan D. (2008). Circulating tumor cells predict survival benefit from treatment in metastatic castration-resistant prostate cancer. Clin. Cancer Res..

[15] Zhou S., Wang L., Zhang W., Liu F., Zhang Y., Jiang B., Wang J., Yuan H. (2021). Circulating Tumor Cells Correlate With Prognosis in Head and Neck Squamous Cell Carcinoma. Technol. Cancer Res. Treat..

[16] Kulasinghe A., Perry C., Jovanovic L., Nelson C., Punyadeera C. (2015). Circulating tumour cells in metastatic head and neck cancers. Int. J. Cancer.

[17] Muller V., Stahmann N., Riethdorf S., Rau T., Zabel T., Goetz A., Janicke F., Pantel K. (2005). Circulating tumor cells in breast cancer: Correlation to bone marrow micrometastases, heterogeneous response to systemic therapy and low proliferative activity. Clin. Cancer Res..

[18] Weller P., Nel I., Hassenkamp P., Gauler T., Schlueter A., Lang S., Dountsop P., Hoffmann A.C., Lehnerdt G. (2014). Detection of circulating tumor cell subpopulations in patients with head and neck squamous cell carcinoma (HNSCC). PLoS ONE.

[19] Pantel K., Alix-Panabieres C. (2010). Circulating tumour cells in cancer patients: Challenges and perspectives. Trends Mol. Med..

[20] Pérez-Barrios C., Nieto-Alcolado I., Torrente M., Jiménez-Sánchez C., Calvo V., Gutierrez-Sanz L., Palka M., Donoso-Navarro E., Provencio M., Romero A. (2016). Comparison of methods for circulating cell-free DNA isolation using blood from cancer patients: Impact on biomarker testing. Transl. Lung Cancer Res..

[21] Cristofanilli M., Broglio K.R., Guarneri V., Jackson S., Fritsche H.A., Islam R., Dawood S., Reuben J.M., Kau S.W., Lara J.M. (2007). Circulating tumor cells in metastatic breast cancer: Biologic staging beyond tumor burden. Clin. Breast Cancer.

[22] Tinhofer I., Staudte S. (2018). Circulating tumor cells as biomarkers in head and neck cancer: Recent advances and future outlook. Expert Rev. Mol. Diagn..

[23] Satelli A., Mitra A., Brownlee Z., Xia X., Bellister S., Overman M.J., Kopetz S., Ellis L.M., Meng Q.H., Li S. (2015). Epithelial-mesenchymal transitioned circulating tumor cells capture for detecting tumor progression. Clin. Cancer Res..

[24] Reisbeck M., Richter L., Helou M.J., Arlinghaus S., Anton B., van Dommelen I., Nitzsche M., Baßler M., Kappes B., Friedrich O. (2018). Hybrid integration of scalable mechanical and magnetophoretic focusing for magnetic flow cytometry. Biosens. Bioelectron..

[25] Gribko A., Kunzel J., Wunsch D., Lu Q., Nagel S.M., Knauer S.K., Stauber R.H., Ding G.B. (2019). Is small smarter? Nanomaterial-based detection and elimination of circulating tumor cells: Current knowledge and perspectives. Int. J. Nanomed..

[26] Siemer S., Wunsch D., Khamis A., Lu Q., Scherberich A., Filippi M., Krafft M.P., Hagemann J., Weiss C., Ding G.B. (2020). Nano Meets Micro-Translational Nanotechnology in Medicine: Nano-Based Applications for Early Tumor Detection and Therapy. Nanomaterials.

[27] Rauscher H., Sokull-Kluttgen B., Stamm H. (2013). The European Commission’s recommendation on the definition of nanomaterial makes an impact. Nanotoxicology.

[28] Shashni B., Ariyasu S., Takeda R., Suzuki T., Shiina S., Akimoto K., Maeda T., Aikawa N., Abe R., Osaki T. (2018). Size-Based Differentiation of Cancer and Normal Cells by a Particle Size Analyzer Assisted by a Cell-Recognition PC Software. Biol. Pharm. Bull..

[29] Nichols A.C., Lowes L.E., Szeto C.C., Basmaji J., Dhaliwal S., Chapeskie C., Todorovic B., Read N., Venkatesan V., Hammond A. (2012). Detection of circulating tumor cells in advanced head and neck cancer using the CellSearch system. Head Neck.

[30] Riethdorf S., Fritsche H., Muller V., Rau T., Schindlbeck C., Rack B., Janni W., Coith C., Beck K., Janicke F. (2007). Detection of circulating tumor cells in peripheral blood of patients with metastatic breast cancer: A validation study of the CellSearch system. Clin. Cancer Res..

[31] Riethdorf S., O’Flaherty L., Hille C., Pantel K. (2018). Clinical applications of the CellSearch platform in cancer patients. Adv. Drug Deliv. Rev..

[32] Obayashi K., Akatsuka J., Endo Y., Takeda H., Hayashi T., Toyama Y., Suzuki Y., Hamasaki T., Kimura G., Ohnaga T. (2019). Initial detection of circulating tumor cells from metastatic prostate cancer patients with a novel small device. Prostate Int..

[33] Boukamp P., Tilgen W., Dzarlieva R.T., Breitkreutz D., Haag D., Riehl R.K., Bohnert A., Fusenig N.E. (1982). Phenotypic and genotypic characteristics of a cell line from a squamous cell carcinoma of human skin. J. Natl. Cancer Inst..

[34] Welkoborsky H.J., Jacob R., Riazimand S.H., Bernauer H.S., Mann W.J. (2003). Molecular biologic characteristics of seven new cell lines of squamous cell carcinomas of the head and neck and comparison to fresh tumor tissue. Oncology.

[35] Gribko A., Hahlbrock A., Strieth S., Becker S., Hagemann J., Deichelbohrer M., Hildebrandt A., Habtemichael N., Wunsch D. (2017). Disease-relevant signalling-pathways in head and neck cancer: Taspase1’s proteolytic activity fine-tunes TFIIA function. Sci. Rep..

[36] Goesswein D., Habtemichael N., Gerhold-Ay A., Mazur J., Wunsch D., Knauer S.K., Kunzel J., Matthias C., Strieth S., Stauber R.H. (2018). Expressional analysis of disease-relevant signalling-pathways in primary tumours and metastasis of head and neck cancers. Sci. Rep..

[37] Wünsch D., Hahlbrock A., Heiselmayer C., Backer S., Heun P., Goesswein D., Stocker W., Schirmeister T., Schneider G., Kramer O.H. (2015). Fly versus man: Evolutionary impairment of nucleolar targeting affects the degradome of Drosophila’s Taspase1. FASEB J..

[38] Deuss E., Gosswein D., Gul D., Zimmer S., Foersch S., Eger C.S., Limburg I., Stauber R.H., Kunzel J. (2020). Growth Factor Receptor Expression in Oropharyngeal Squamous Cell Cancer: Her1-4 and c-Met in Conjunction with the Clinical Features and Human Papillomavirus (p16) Status. Cancers.

[39] Barak V., Goike H., Panaretakis K.W., Einarsson R. (2004). Clinical utility of cytokeratins as tumor markers. Clin. Biochem..

[40] Foddai A., Elliott C.T., Grant I.R. (2010). Maximizing capture efficiency and specificity of magnetic separation for Mycobacterium avium subsp. paratuberculosis cells. Appl. Environ. Microbiol..

[41] Foddai A.C.G., Grant I.R. (2020). A novel one-day phage-based test for rapid detection and enumeration of viable Mycobacterium avium subsp. paratuberculosis in cows’ milk. Appl. Microbiol. Biotechnol..

[42] Siemer S., Westmeier D., Barz M., Eckrich J., Wunsch D., Seckert C., Thyssen C., Schilling O., Hasenberg M., Pang C. (2018). Biomolecule-corona formation confers resistance of bacteria to nanoparticle-induced killing: Implications for the design of improved nanoantibiotics. Biomaterials.

[43] Hussain T., Gellrich D., Siemer S., Reichel C.A., Eckrich J., Dietrich D., Knauer S.K., Stauber R.H., Strieth S. (2021). TNF-alpha-Inhibition Improves the Biocompatibility of Porous Polyethylene Implants In Vivo. Tissue Eng. Regen. Med..

[44] Westmeier D., Siemer S., Vallet C., Steinmann J., Docter D., Buer J., Knauer S.K., Stauber R.H. (2020). Boosting nanotoxicity to combat multidrug-resistant bacteria in pathophysiological environments. Nanoscale Adv..

[45] Stauber R.H., Siemer S., Becker S., Ding G.B., Strieth S., Knauer S.K. (2018). Small Meets Smaller: Effects of Nanomaterials on Microbial Biology, Pathology, and Ecology. ACS Nano.

[46] Stauber R.H., Westmeier D., Wandrey M., Becker S., Docter D., Ding G.B., Thines E., Knauer S.K., Siemer S. (2020). Mechanisms of nanotoxicity—Biomolecule coronas protect pathological fungi against nanoparticle-based eradication. Nanotoxicology.

[47] Sieuwerts A.M., Kraan J., Bolt-de Vries J., van der Spoel P., Mostert B., Martens J.W., Gratama J.W., Sleijfer S., Foekens J.A. (2009). Molecular characterization of circulating tumor cells in large quantities of contaminating leukocytes by a multiplex real-time PCR. Breast Cancer Res. Treat..

[48] Chudziak J., Burt D.J., Mohan S., Rothwell D.G., Mesquita B., Antonello J., Dalby S., Ayub M., Priest L., Carter L. (2016). Clinical evaluation of a novel microfluidic device for epitope-independent enrichment of circulating tumour cells in patients with small cell lung cancer. Analyst.

[49] Ciccioli M., Bravo-Santano N., Davis A., Lewis J., Malcolm R., Pailhes-Jimenez A.S. Mesenchymal markers: The new avenue for circulating tumor cells detection. Proceedings of the ANGLE plc AACR 2021.

[50] Grover P.K., Cummins A.G., Price T.J., Roberts-Thomson I.C., Hardingham J.E. (2014). Circulating tumour cells: The evolving concept and the inadequacy of their enrichment by EpCAM-based methodology for basic and clinical cancer research. Ann. Oncol..

[51] Grisanti S., Almici C., Consoli F., Buglione M., Verardi R., Bolzoni-Villaret A., Bianchetti A., Ciccarese C., Mangoni M., Ferrari L. (2014). Circulating tumor cells in patients with recurrent or metastatic head and neck carcinoma: Prognostic and predictive significance. PLoS ONE.

[52] Menarini Silicon Biosystems Inc. https://documents.cellsearchctc.com/pdf/e631600006/e631600006_EN.pdf.

