# Supplementary material for: IsoMAG—An Automated System for the Immunomagnetic Isolation of Squamous Cell Carcinoma-Derived Circulating Tumor Cells
Source: Diagnostics (Basel). 2021 Nov 4;11(11):2040. doi: 10.3390/diagnostics11112040 (PMC8623084; doi:10.3390/diagnostics11112040)
Supplement: Supplementary file 1 [file diagnostics-11-02040-s001.zip › diagnostics-1408314-supplementary.pdf]

Supplementary

IsoMAG – an automated system for the immunomagnetic isolation of squamous cell carcinoma-derived circulating tumor cells

Alena Gribko [1,†], Janis Stiefel [2,†], Lana Liebetanz [2], Sophie Madeleine Nagel [1], Julian Künzel [3], Madita Wandrey [1], Jan Hagemann [1], Roland H. Stauber [1], Christian Freese [2,*] and Désirée Gül [1,*]

[1] Department of Otorhinolaryngology/ENT University Medical Center Mainz, Langenbeckstr. 1, 55131 Mainz, Germany
[2] Fraunhofer Institute for Microengineering and Microsystems IMM, Carl-Zeiss-Str. 18-20, 55129 Mainz, Germany
[3] Department of Otorhinolaryngology, University Hospital Regensburg, Franz-Josef-Strauß-Allee 11, 93053 Regensburg, Germany
* Correspondence: Christian.freese@imm.fraunhofer.de; guel@uni-mainz.de
† Contributed equally

Tables

Table S1. Detailed protocol of IsoMAG isolation procedure

No.	Program section	Chapter title	Carousel: position	Magnet: position	Flowrate [ml/min]	Duration [s]
**1**	Initialization	Home position	Position 1	Position home	40	
**2**		to pipette position				
**3**		Taking pipette				
**4**		Pipette up				
**5**		to carousel position				
**6**	Blood sample	to blood sample position				
**7**		Blood sample mixing			10	1800
**8**		Aspirate blood			10	
**9**		Aspirate to extra position				
**10**		Aspirate to carousel position				
**11**		Waiting				5
**12**		magnet pos 1		Position 1		200
**13**		magnet pos 2		Position 2		400

**14**		waste blood			1	
**15**	Washing buffer 1<br>5ml	pipette up/ change to position 2	Position 2	Position home		
**16**		wash buffer mixing			10	600
**17**		wash buffer				
**18**		Aspirate			10	
**19**		Aspirate to extra position				
**20**		cone mixing			10	60
**21**		Aspirate buffer			10	
**22**		Aspirate to carousel position				
**23**		magnet pos 1		Position 1		200
**24**		magnet pos 2		Position 2		400
**25**		waste buffer			1	
**26**	Washing buffer 2<br>5ml	pipette up/change to position 3	Position 3	Position home		
**27**		wash buffer mixing			10	600
**28**		wash buffer				
**29**		Aspirate			10	
**30**		Aspirate to extra position				
**31**		cone mixing			10	60
**32**		Aspirate buffer			10	
**33**		Aspirate to carousel position				
**34**		magnet pos 1		Position 1		200
**35**		magnet pos 2		Position 2		400
**36**		waste buffer			1	
**37**	Washing buffer 3<br>4ml	pipette up/change to position 4	Position 4	Position home		
**38**		4ml wash buffer mixing			10	600
**39**		wash buffer				
**40**		Aspirate			10	
**41**		Aspirate to extra position				
**42**		cone mixing			10	60
**43**		Aspirate buffer			10	
**44**		Aspirate to carousel position				

**45**		magnet pos 1		Position 1		200
**46**		waste buffer			1	
**47**		waiting				200
**48**		waste buffer 2			1	
**49**	Washing buffer 4<br>1ml	pipette up/change position 5	Position 5	Position home		
**50**		wash buffer mixing			10	300
**51**		wash buffer				
**52**		Aspirate			5	
**53**		Aspirate to carousel position				
**54**		pipette up				
**55**		eppi dialog				1
**56**		dispense liquid			5	
**57**		Home position + removing falcons and pipette				

Figures

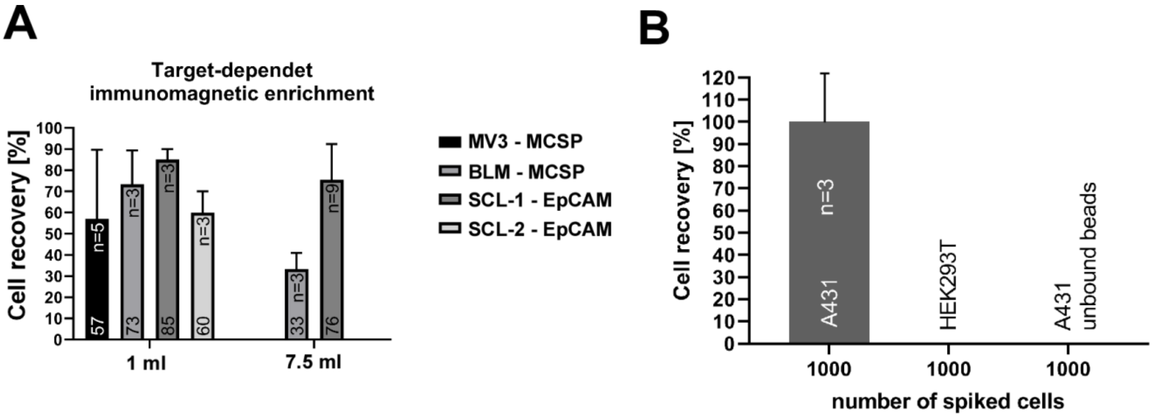

Figure S1. Recovery rates of different cancer cell lines during protocol establishment. **A**. Melanoma and carcinoma single cells were recovered from 1 and 7.5 mL culture medium. 20 CFSE labeled single cell were spiked in 1 or 7.5 mL respectively and manually enriched using a magnetic separator. Dynabeads MyOne Streptavidin T1 beads were coated with 20 µg/mL biotinylated monoclonal mouse anti-human melanoma-associated chondroitin sulfate proteoglycan (MCSP) antibody EP-1 (Miltenyi Biotec) for melanoma cells MV3 and BLM or biotinylated monoclonal mouse anti-human EpCAM (CD326) antibody 1B7 for squamous cell carcinoma cells SCL-1 and SCL-2. **B**. A431 cells (EpCAM-positive) and HEK293T cells (negative control) were spiked into 7.5 mL culture medium and enriched as described in A.

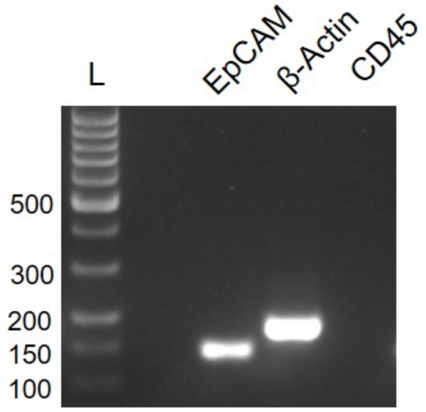

Figure S2. Gel electrophoresis of EpCAM RT-qPCR products (136 bp) from SCL-1 single cell RNA. Total single cell RNA was isolated using RNeasy Micro Kit (QIAGEN) with a downscaled protocol spiking one cell in lysis buffer and reverse-transcribed in cDNA with Sensifast cDNA synthesis Kit (Bioline). qPCR was performed with Quantifast qPCR Kit (QIAGEN). β-Actin (176 bp) served as positive control and leukocyte marker CD45 (159 bp) was used as negative control. Primers were designed with Primer-BLAST (National Institutes of Health; https://www.ncbi.nlm.nih.gov/tools/primer-blast/).

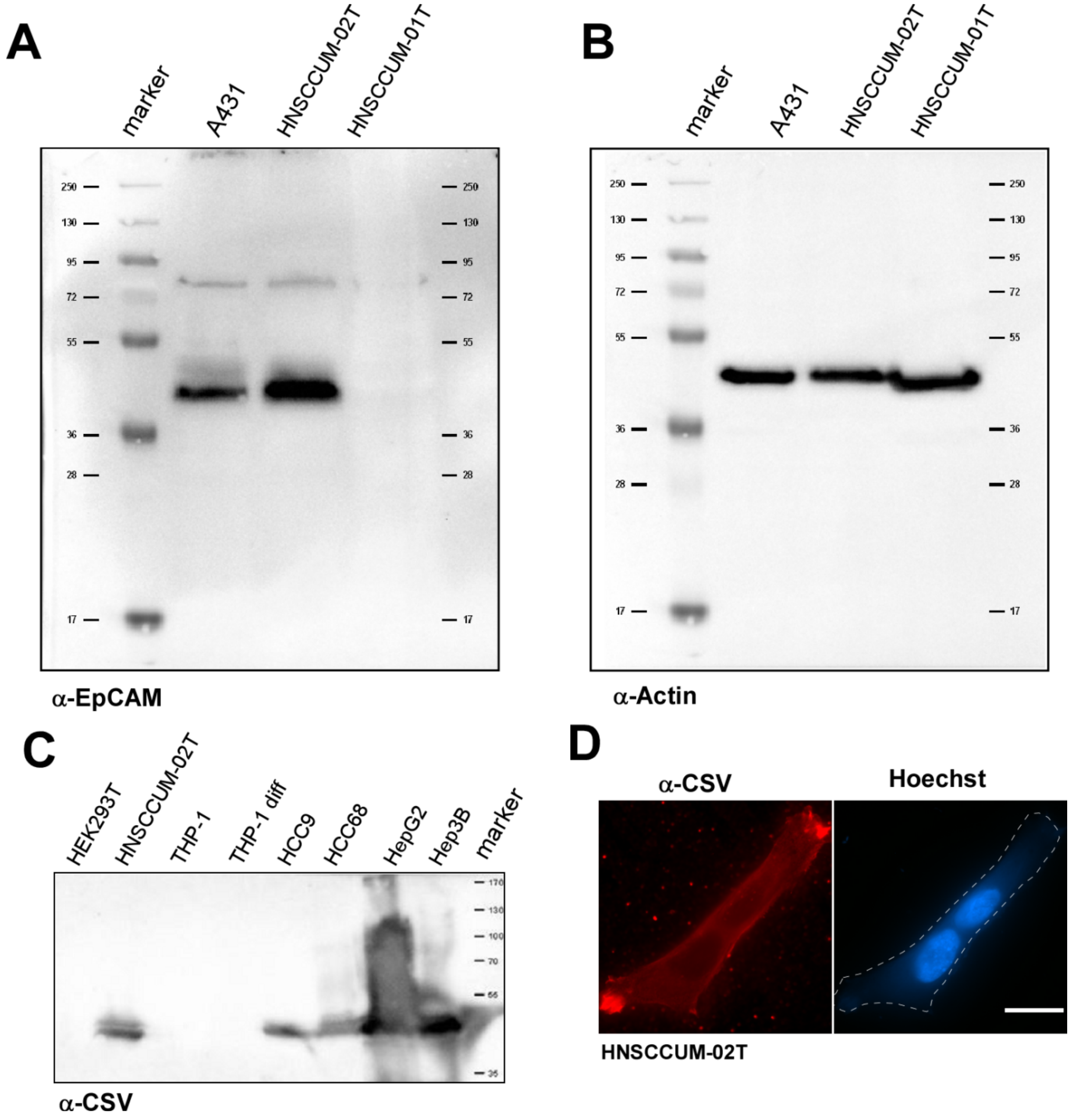

Figure S3. Expression of EpCAM protein (**A**, **B**) and CSV (**C**, **D**) in HNSCCUM-02T. Expression of EpCAM protein in whole cell lysates was visualized using α-CD326 purified Ab (44 kDa, eBioscience #14-9326) and α-Actin Ab (42 kDa, Sigma #A2066) served as loading control (**B**). Expression of CSV protein was visualized using α-cell surface vimentin Ab (50 kDa, Abnova, 17121-84-1) by Western Blot of whole cell lysates (**C**) and immunofluorescence staining of HNSCCUM-02T cells (**D**). Nuclei were stained with Hoechst. Scale bar, 10 μm.

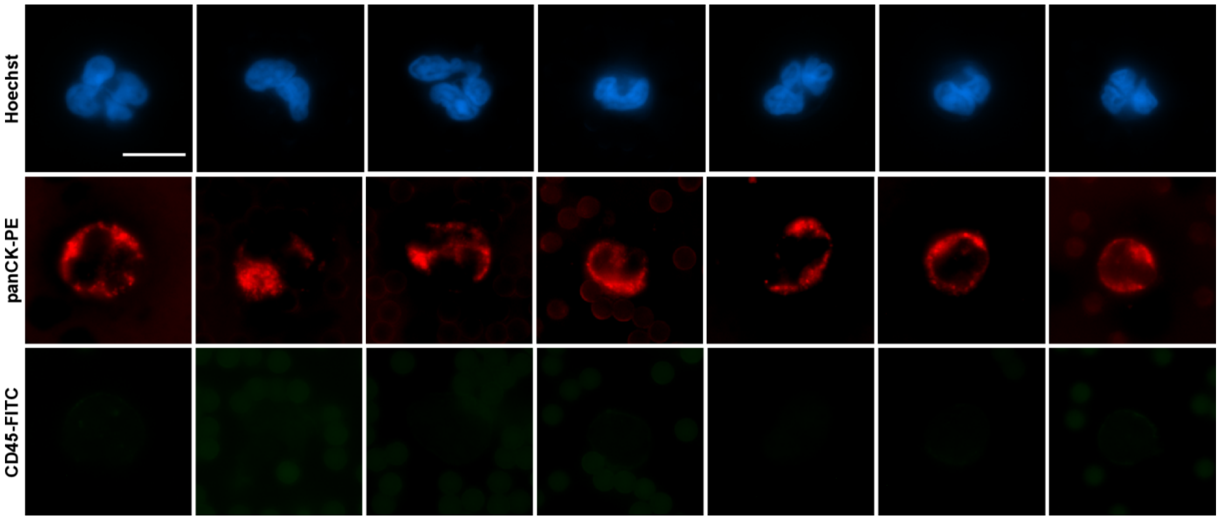

Figure S4. Potential circulating tumor cells (CTCs) could be enriched from whole blood of a HNSCC patient using EpCAM and CSV beads. 7.5 mL of patient blood was mixed with EpCAM- and CSV-coupled T1 beads (1:1) and placed into the IsoMAG unit for automated enrichment of CTCs. For cell counting, enriched cells were stained with Hoechst dye, cytokeratin (panCK-PE), and CD45-FITC antibodies and quantified by fluorescence microscopy. [Hoechst+/CD45-/panCK+] cells were classified as potential CTCs. Scale bar, 10 μm.

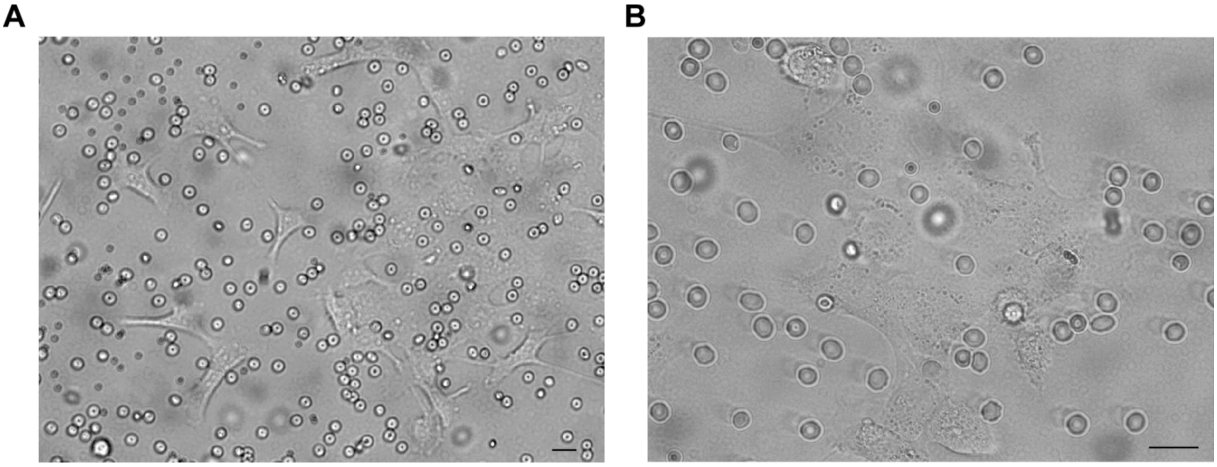

Figure S5. Brightfield microscopic images (20-fold (**A**) and 40-fold (**B**) magnification) of HNSCCUM-02T cells which were cultivated after immunomagnetic isolation using the Dynabeads™ FlowComp™ Flexi Kit (Invitrogen). 35 HNSCCUM-02T cells were spiked into 7.5ml blood of healthy donor, and manually isolated using EpCAM beads as described in Materials and Methods section. Additionally, after repeated magnetic separation and washing steps cells were re-suspended in 1ml release buffer and incubated for 10min on ice. The supernatant was centrifuged (350xg, 10min), and the cell pellet was resuspended in a mixture of fresh and pre-conditioned medium (1/3 to 2/3). Cells were seeded in collagen slides (#80802, ibidi GmbH, Gräfelfing, Germany), and imaged 7 days after seeding. Scale bars, 20 μm.

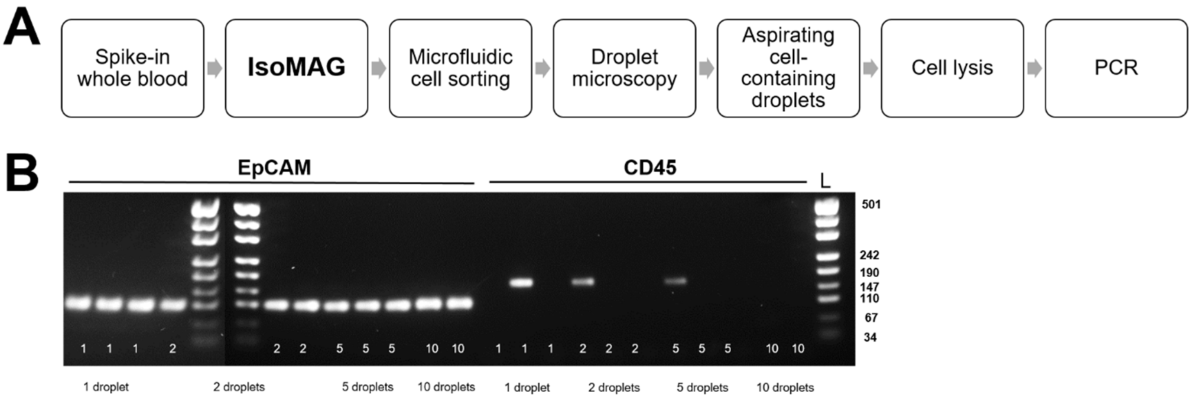

C

#droplets/sample	n	EpCAM+ [%] (n)	CD45+ [%] (n)	Purity [%] (#droplets)
1	3	100 (3)	33 (1)	66
2	3	100 (3)	≤ 33 (1)	≥ 66
5	3	100 (3)	≤ 33 (1)	≥ 66
10	2	100 (2)	0 (0)	100
**Total**	**11**	**100 % (11)**	**≤ 25 % (3)**	**≥ 75 %**

Figure S6. Establishment of CTCelect unit improves purity of isolated tumor cells from whole blood. **A**. Workflow of cell analysis including IsoMAG isolation unit, microfluidic cell sorting, and downstream PCR analysis. CFSE stained tumor cells (MCF-7) were spiked in 7.5 mL whole blood from a healthy donor. Immunomagnetic pre-enrichment using IsoMAG reduced WBC concentration by 10,000-fold compared to whole blood enabling microfluidic cell sorting. We used an in-house developed cell sorting unit consisting of a microfluidic chip for hydrodynamic sample focusing and a 480 nm laser with two silicium photomultiplier-supported fluorescence detectors to dispense single cells in single droplets. By establishing appropriate flow conditions and droplet sizes in chip-based microfluidic systems, the arithmetical probability of dispensing one WBC with a cancer cell in the same droplet is 10 %. Droplets containing a fluorescent tumor cell were identified under the fluorescence microscope and pooled in samples of 1, 2, 5 (*n*=3) and 10 (*n*=2) droplets. Samples were lysed for 15 min at 4 °C using a guanidine salt-free lysis buffer for direct one-step RT-PCR with the SensiFAST™ SYBR No-ROX One-Step Kit (Bioline) according to the manual. **B**. RT-PCR products were analyzed by means of gel electrophoresis. Leukocyte marker CD45 (159 bp) was targeted to detect WBCs in the droplets. EpCAM transcript (136 bp) served as positive control for tumor cells. Primers were designed with Primer-BLAST (National Institutes of Health; https://www.ncbi.nlm.nih.gov/tools/primer-blast/). **C.** WBC contamination was quantified as the ratio between samples positive for CD45 and EpCAM. Depending on the sample size, only 0-33 % (mean ≤ 25 %) of the samples were CD45+. Consequently, a purity of at least 75 % was determined and the feasibility of downstream PCR analysis after automated immunomagnetic enrichment and cell sorting was confirmed.
